# Physical Activity Among Rural Men: Barriers and Preferences

**DOI:** 10.5888/pcd20.230046

**Published:** 2023-10-05

**Authors:** Jacob Gallagher, Emine O. Bayman, Lisa A. Cadmus-Bertram, Nathaniel D.M. Jenkins, Amy Pearlman, Kara M. Whitaker, Lucas J. Carr

**Affiliations:** 1 Department of Health and Human Physiology, University of Iowa, Iowa City; 2 Departments of Biostatistics and Anesthesia, University of Iowa, Iowa City; 3 Department of Kinesiology, University of Wisconsin, Madison; 4 Prime Institute, Miami, Florida; 5 Department of Epidemiology, University of Iowa, Iowa City

## Abstract

**Introduction:**

Physical activity positively affects health. Although 94% of Americans know the health benefits of regular physical activity, more than 75% do not achieve recommended levels. The objective of our study was to identify and define the key components of a physical activity intervention tailored to rural American men.

**Methods:**

We recruited rural men (N = 447) via Amazon’s Mechanical Turk online platform to complete a needs assessment survey focused on their interest in a physical activity intervention, preferred intervention features, and potential intervention objectives. Data were summarized by using descriptive statistics. A cumulative logistic regression model examined associations between the men’s perceived importance of physical activity to health and their interest in a physical activity intervention.

**Results:**

Almost all participants (97.7%) rated physical activity as “at least somewhat important” to their health, and 83.9% indicated they would be “at least somewhat interested” in participating in a physical activity intervention. On a scale of 1 (not at all a barrier) to 5 (very much a barrier), motivation (mean 3.4; 95% CI, 3.3–3.5), cold weather (mean, 3.4; 95% CI, 3.3–3.5), and tiredness (mean, 3.3; 95% CI, 3.2–3.4) were rated the biggest barriers to physical activity. Becoming fitter (54.1%) was the top reason for joining a physical activity program. Preferred delivery channels for receiving an intervention were mobile application (ranked from 1 being the most preferred and 9 being the least preferred: mean, 2.8; 95% CI, 2.70–3.09) and e-mail (mean, 4.2; 95% CI, 3.92–4.36). Rural men preferred interventions that taught them how to exercise and that could be done from home.

**Conclusion:**

Our findings suggest US men in rural areas are receptive to physical activity programs. A systematic approach and a clear model of development are needed to tailor future physical activity interventions to the special needs of rural men.

**Summary**

**What is already known on this topic?**

Rural men are underrepresented in physical activity interventions and are at higher disease risk than urban men.

**What is added by this report?**

We surveyed a sample of rural men to determine barriers to physical activity and preferences for interventions.

**What are the implications for public health practice?**

A systematic approach and a clear model of development are needed to tailor physical activity interventions to the special needs of rural men.

## Introduction

Physical activity positively affects health (1). An estimated 94% of Americans know the health benefits of regular physical activity (2), but more than 75% do not achieve recommended activity levels (3). Almost 25% of Americans who do not meet recommended levels report not participating in any leisure-time physical activity (3). These findings suggest that information alone is not enough to make a healthy behavior change (4) and that interventions are needed that promote more than awareness of the benefits of physical activity.

The 23 million US men who live in rural areas are an understudied population at increased risk for inactivity-related chronic conditions. For example, rural men have a 19% higher age-adjusted death rate than urban men and a 39% higher rate than rural women (5). They also have higher rates of inactivity-related chronic conditions, such as cardiovascular disease (6), diabetes (7), cancer (8), and obesity (3). These differences may be due in part to observed differences in physical activity rates because fewer rural men report meeting physical activity guidelines compared with urban men (9). Although increasing physical activity rates among rural men could reduce the gap in urban and rural health disparities (1), rural men are underrepresented in physical activity interventions (10,11). Behavioral interventions are needed to increase physical activity in this population to improve their health outcomes.

The Obesity-Related Behavioral Intervention Trials (ORBIT) model (12) was designed by a working group from the National Institutes of Health (including the National Cancer Institute and the National Heart, Lung, and Blood Institute) to facilitate and standardize the development of behavioral interventions. The ORBIT model emphasizes understanding the needs of a study population before tailoring an intervention to that population’s needs. Such a systematic approach is more effective than nonsystematic approaches. However, little information is currently available on the physical activity-related needs of American rural men. Therefore, the primary aim of our study was to identify the key components and objectives of a physical activity intervention tailored to the unique needs of rural US men to inform the development of future interventions directed at them.

## Methods

### Participants

We recruited a nationwide sample of rural residents (N = 447), who identified through a 2-item eligibility screener as male and living in a rural area. We defined rural according to the US Department of Agriculture’s definition as a town with fewer than 2,500 residents or an area outside a town (13).

Study participants were recruited from among US participants in Amazon’s Mechanical Turk (MTurk), an online crowdsourcing platform. A full description of MTurk is available on their website (mturk.com). We advertised our study on MTurk in broad terms as “A study on physical activity” so that MTurk workers (ie, users who sign up and get paid to complete small tasks) did not know our eligibility criteria (men residing in rural areas) before answering gender and residence (rural or urban) questions. Interested workers were directed to an online survey hosted on the Qualtrics software platform (Qualtrics XM).

We obtained informed consent from all participants before they completed our survey. To be included in the final data analysis, participants had to respond correctly to an attention-check question to ensure data quality. Less than 5% of participant data were missing (ie, missed questions or nonresponses). Settings were configured to prevent people from retaking the survey, using automated systems, or completing the survey too quickly. The Social Sciences Institutional Review Board at the University of Iowa approved all study procedures.

### Research design

The first phase of the ORBIT model focuses on identifying appropriate intervention techniques (ie, reasons for joining, delivery methods, program features) and potential participants in the intervention (14). Consistent with the ORBIT model, our study used a cross-sectional design with participants completing a single survey that addressed 3 main areas: 1) interest in a physical activity intervention, 2) preferred intervention features, and 3) potential intervention goals. The survey was modeled after Cadmus-Bertram’s 2019 mail-based survey, which asked about physical activity barriers and facilitators among rural women residing in Wisconsin (15). On completion of the survey, participants were given a completion code to enter on MTurk to receive $2.00.

### Demographics

Participants were asked to report their age, ethnicity, race, education level, income, marital status, number of children under the age of 18 in the household, perceived health status (excellent, very good, good, fair, poor), employment status (full-time; part-time; unemployed, looking for work; unemployed, not looking for work; full-time or part-time student; disabled; retired), occupation, and a basic description of physical activity the occupation entailed (mostly sitting or standing, mostly walking, mostly heavy labor or physically demanding work).

### Interest in a physical activity intervention

Participants ranked how important they believed physical activity was for good health on a 5-point Likert scale from 1 (not at all important) to 5 (very important). Participants also rated on a 5-point Likert scale from 1 (not at all interested) to 5 (very interested) how interested they would be in a program that could help them be more active. 

### Preferred intervention techniques

Access to and use of computer technology were assessed to determine how best to deliver future interventions to rural men. Participants reported their access to the internet (eg, broadband, dial-up) and to cellular telephone service (smartphone, traditional [does only SMS texting and telephone calls], or no cellular telephone). Participants answered yes or no to a question that asked what they used their telephone for (calls, internet, applications). Participants also reported their use of wearable physical activity trackers (eg, Fitbits, pedometers). Participants answered yes or no to the question about whether, to be physically active, they had used or were willing to try various online resources (eg, exercise videos), internet-connected devices (eg, Peloton spin bikes), or telehealth services.

We then assessed facilitators to physical activity and participation in interventions by asking participants about their preferred types of exercise. We also asked about their preferred delivery method for a physical activity program with a 9-item list of delivery methods: video conferencing, telephone, group training, in-person one-on-one, mail, social media, text messaging, email, or mobile app. Environmental facilitators and barriers were assessed by asking what facilities were currently available and whether they used those facilities. The survey also asked about reasons for joining a physical activity program and whom they might be interested in being active with (eg, significant others, friends, coworkers). Participants selected whether certain program features (eg, “can be done from home,” “men only”) were required to partake in the program or were barriers to participating in a program.

### Potential intervention objectives

Addressing common barriers to physical activity is one way for an intervention to promote physical activity. To understand the barriers rural men perceive to physical activity, participants were asked to rank on a Likert scale from 1 (not at all a barrier) to 5 (very much a barrier) the degree to which 25 commonly cited barriers to physical activity interfered with their physical activity behavior (eg, lack of motivation, weather, fear of exposure to COVID-19) (15). 

### Statistical analysis

To generalize to the 23 million rural men in the US we needed an estimated sample size of at least 385 participants to have a 5% margin of error using 95% CIs. To summarize data we used descriptive statistics, including mean and standard deviation for continuous variables and frequencies and percentages for categorical variables. We used the VGAM (Vector Generalized Linear and Additive Models) package in R (16) to analyze the multivariable associations of the importance of physical activity to health and the likelihood of being interested in a physical activity intervention by a cumulative logistic regression model. Data analysis was performed by using R (R Foundation for Statistical Computing).

## Results

### Demographics

A total of 8,182 MTurk workers completed our 2-item screener. Of these, 4,535 did not identify as male: 4,402 responded female, 61 responded nonbinary, 53 preferred not to answer, and 19 gave no response. Of the remaining male respondents, 3,039 did not live in a rural community. Another 161 who took the survey and otherwise qualified did not pass the attention check. After screening out those who did not meet our eligibility criteria, 447 rural men were included in our study. 

Participants were mostly White (85.5%), non-Hispanic (84.6%), highly educated (79.3% had a college degree), and married (72.5%) (Table 1) with an average age of 34.7 years (SD, 11.7). By using IP addresses to determine geographic region as defined by the US Census Bureau, we determined that 125 (28%) participants were from the Midwest, 48 (11%) from the Northeast, 139 (31%) from the South, and 108 (24%) from the West. We were unable to identify 27 participants by IP address; these respondents were included as “Unknown/unsure” of location.

**Table 1:** Demographic Characteristics Reported by a Nationwide Sample (N = 447) of Rural US Men

Characteristic	Percentage
**Ethnicity**	
Not Hispanic or Latino	84.6
Hispanic or Latino	14.5
**Race**	
Black/African American	6.7
White	85.5
**Education**	
High school graduate	7.6
Some college	12.1
Trade, technical, or vocational training	2.9
College graduate	40.0
Some post-graduate work	8.9
Post-graduate degree	27.5
**Annual income, $**	
<24,999	12.1
25,000–49,999	22.6
50,000–74,999	22.4
75,000–99,999	25.3
100,000–149,999	14.6
>150,000	2.9
**No. of children**	
0	31.8
1	33.8
2	29.3
≥3	4.7
**Marital status**	
Married	72.5
Never married	22.8
Divorced	2.2
**Self- reported health status**	
Excellent	40.3
Very good	34.3
Good	24.7
Poor	0.7
**Employment status**	
Full-time	79.2
Looking for work	7.2
Student	3.4
Retired	3.4
**Occupational activity**	
Mostly sitting or standing	62.2
Mostly walking	19.9
Mostly heavy labor or physical demanding work	14.5

### Interest in a physical activity intervention

A total of 97.7% of rural men in our sample viewed exercise as at least somewhat important to their health, and 40.3% reported it as very important. Overall, most reported interest (83.9%) in joining a physical activity program; 23.9% were very interested, 33.6% were interested, and 26.4% were somewhat interested. The more important that men viewed exercise to be to their health, the more likely they were to be at least somewhat interested in a physical activity program. Each category increase for importance (ie, somewhat important to important) was associated with 1.38 times (odds ratio [OR] = 1.38; 95% CI, 1.13–1.68) greater odds of being more interested in a physical activity program (ie, greater odds in very interested compared with interested, or of interested compared with somewhat interested). Breaking out responses by demographic characteristics, we found substantial variation in levels of interest (Appendix).

### Preferred intervention techniques

Most rural men in our sample (77.6%) reported using broadband and 11.9% used only their cellular telephones for internet access. For cellular telephone service, 86.1% used smartphones, and 11.0% used a traditional cellular telephone. The remaining participants did not use a cellular telephone (2.9%). For smartphone users, common uses were texting (99.7%), photographs (96.3%), email (95.0%), internet (92.5%), social media (91.9%), calendar (83.0%), and video conferencing (68.2%).

For remote resources, more than half of men in our sample (60.1%) currently used exercise smartphone applications or had used them in the past, and 52.2% had used exercise training videos (eg, P90x, Beach Body). Respondents had less experience with online exercise classes (49.3% currently used or had used them previously), internet-connected devices (44.9%; eg, Peloton), pedometers (40.5%), smartwatches (40.3%), and Fitbits (38.6%). Among the men who reported not using these systems in the past, 68.0% were interested in trying online exercise classes, 62.1% in internet-connected devices, 59.1% in smartphone exercise applications, 49.2% in exercise training videos, 48.3% in smartwatches, 45.3% in Fitbits, 44.8% in digital scales, and 42.9% in pedometers.

**Figure 1:**
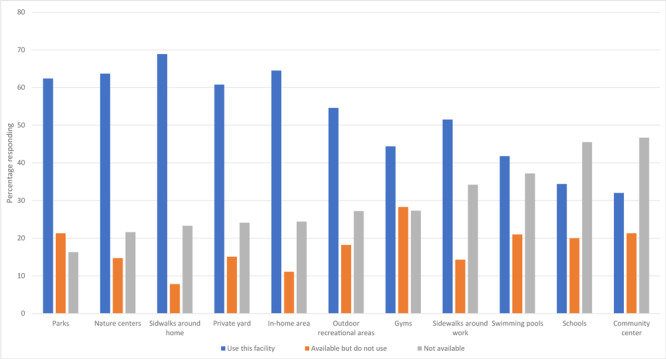
Facilities or locations for physical activity available to a nationwide sample (N = 447) of rural US men and percentage who used them.

Although 84% of participants reported that parks were the most available facility for physical activity, 68.9% said they used the sidewalks around their home (Figure 1). For social support, participants reported being active with friends (64.2%), children (50.1%), or a significant other (49.2%). Fewer men were interested in being active with other community members (31.3%), coworkers (30.2%), family (28.2%), exercise groups (23.5%), pets (19.0%), and other community members (2.9%).

**Table:** 

Exercise facility or location	Use this	Available but do not use	Not available
Parks	62.4	21.3	16.3
Nature centers	63.7	14.7	21.6
Sidewalks around home	68.9	7.8	23.3
Private yard	60.8	15.1	24.1
In-home area	64.5	11.1	24.4
Outdoor recreational areas	54.6	18.2	27.2
Gyms	44.4	28.3	27.3
Sidewalks around work	51.5	14.3	34.2
Swimming pools	41.8	21.0	37.2
Schools	34.4	20.0	45.5
Community center	32.0	21.3	46.7

Respondents reported that the following were 3 of their favorite types of physical activity: walking (64.4%), running (51.7%), and biking (40.7%). Men who said they were interested in a physical activity program gave the following as their reasons: to be more physically fit (54.1%), to get more energy (52.6%), and to improve mood or mental health (45.2%). When asked to rank how they would prefer to receive a physical activity program, the highest-ranked of the 9 options were mobile applications (mean, 2.8; 95% CI, 2.70–3.09), e-mail (mean, 4.2; 95% CI, 3.92–4.36), social media (mean, 4.6; 95% CI, 4.47–4.89), and text messaging (mean, 4.6; 95% CI, 4.38–4.78.). Video conferencing (mean, 6.7; 95% CI, 6.51-7.00), telephone (mean, 6.0; 95% CI, 5.85–6.23), and in-person (mean, 5.5; 95% CI, 5.22–5.68) were ranked the lowest.

**Figure 2:**
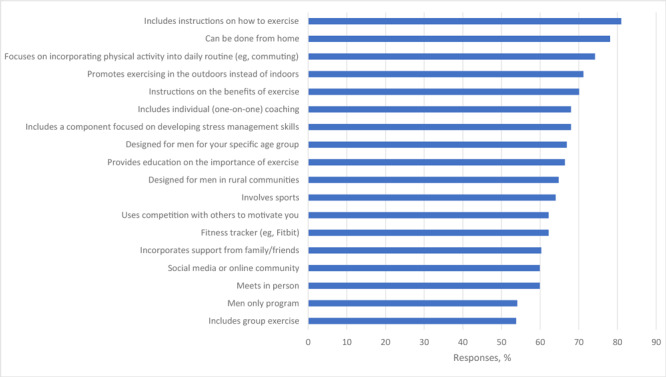
Selected features preferred or required in a physical activity program by a nationwide sample (N = 447) of rural US men.

When asked which program features they preferred in a physical activity program, responses were instructions on how to exercise (81.0% of participants), exercises that can be done from home (78.1%), and programs that focus on incorporating physical activity into a daily routine (74.2%) (Figure 2).

**Table:** 

Feature	%
Includes instructions on how to exercise	81.0
Can be done from home	78.1
Focuses on incorporating physical activity into daily routine (eg, commuting)	74.2
Promotes exercising in the outdoors instead of indoors	71.2
Provides instructions on the benefits of exercise	70.1
Includes a component focused on developing stress management skills or mindfulness	68.0
Includes individual (one-on-one) coaching	68.0
Designed for men for my specific age group	66.9
Provides education on the importance of exercise	66.4
Designed for men in rural communities	64.8
Involves sports	64.0
Uses fitness tracker (eg, Fitbit)	62.2
Uses competition with others to motivate	62.2
Incorporates support from family or friends	60.3
Conducted in person	59.9
Conducted via social media or online community	59.9
Men-only program	54.1
Includes group exercise	53.8

### Potential intervention targets

The most challenging barriers to physical activity among rural men, rated on a scale of 1 (not at all a barrier) to 5 (very much a barrier), were motivation (mean, 3.4; 95% CI, 3.3–3.5), cold weather (mean, 3.4; 95% CI, 3.3–3.5), and tiredness (mean, 3.3; 95% CI, 3.2–3.4) (Table 2).

**Table 2:** Barriers to Physical Activity Reported by a Nationwide Sample (N = 447) of Rural US Men, by Region^a^

Barrier	All	Region			
		Northeast (n = 48)	Midwest (n = 125)	South (n = 139)	West (n = 108)
Motivation	3.4 (3.3–3.5)	3.3 (2.9–3.7)	3.3 (3.1–3.5)	3.5 (3.3–3.7)	3.5 (3.2–3.7)
Cold weather	3.4 (3.3–3.5)	2.9 (2.6–3.3)^b^	3.3 (3.1–3.5)	3.5 (3.3–3.7)	3.5 (3.3–3.7)
Tiredness	3.3 (3.2–3.4)	3.2 (2.8–3.5)	3.2 (3.0–3.4)	3.4 (3.2–3.6)	3.3 (3.1–3.5)
Hard to find time because of housework	3.2 (3.1–3.3)	3.3 (2.9–3.6)	3.0 (2.7–3.2)	3.2 (3.0–3.4)	3.4 (3.2–3.6)
Hard to find time because of job	3.2 (3.0–3.3)	3.0 (2.6–3.4)	3.0 (2.7–3.2)	3.2 (3.0–3.5)	3.3 (3.1–3.5)
Weather issues in the heat	3.1 (3.0–3.2)	2.6 (2.1–3.0)^b^	2.8 (2.6–3.0)^b^	3.2 (3.0–3.5)	3.4 (3.2–3.6)
Fear for safety (COVID-19)	3.1 (3.0–3.2)	2.7 (2.2–3.1)	2.8 (2.5–3.1)	3.1 (2.9–3.4)	3.6 (3.4–3.8)^b^
Short daylight hours in the winter	3.0 (2.8–3.1)	2.6 (2.2–3.0)	2.9 (2.6–3.1)	2.9 (2.6–3.1)	3.4 (3.2–3.6)^b^
No convenient places to exercise indoors	3.0 (2.8–3.1)	2.5 (2.1–3.0)	2.8 (2.6–3.1)	3.0 (2.7–3.2)	3.3 (3.1–3.5)^b^
Health problems make it hard to be active	2.9 (2.7–3.0)	2.4 (2.0–2.9)	2.7 (2.5–3.0)	2.9 (2.7–3.2)	3.2 (3.0–3.5)
Cost	2.9 (2.7–3.0)	2.2 (1.8–2.6)	2.7 (2.4–3.0)	2.9 (2.6–3.1)	3.4 (3.2–3.6)^b^
Dislike physical activity	2.8 (2.7–3.0)	2.5 (2.1–2.9)	2.8 (2.5–3.0)	2.8 (2.5–3.0)	3.2 (3.0–3.5)^b^
No convenient place to exercise outdoors	2.8 (2.7–3.0)	2.4 (2.0–2.8)	2.8 (2.5–3.0)	2.8 (2.6–3.0)	3.2 (3.0–3.4)^b^
I don't have anyone to exercise with	2.8 (2.7–2.9)	2.6 (2.2–3.0)	2.5 (2.3–2.8)	2.8 (2.6–3.0)	3.2 (2.9–3.4)^b^
Unsure how to get started	2.8 (2.6–2.9)	2.4 (2.0–2.7)	2.7 (2.4–2.9)	2.7 (2.5–3.0)	3.2 (3.0–3.4)^b^
Fear of injury	2.7 (2.6–2.9)	2.2 (1.8–2.7)	2.5 (2.2–2.7)	2.8 (2.5–3.0)	3.4 (3.1–3.6)^b^
Lack of support from family or spouse	2.7 (2.6–2.8)	2.1 (1.7–2.5)^b^	2.5 (2.3–2.8)^b^	2.8 (2.6–3.0)^b^	3.1 (2.9–3. 4)^b^
Hard to find time due to caregiving for a child	2.7 (2.6–2.8)	2.5 (2.0–2.9)	2.6 (2.4–2.9)	2.6 (2.3–2.8)	3.2 (3.0–3.4)^b^
Fear for safety because of traffic	2.7 (2.5–2.8)	2.1 (1.7–2.5)	2.5 (2.2–2.8)	2.7 (2.5–3.0)	3.3 (3.1–3.5)^b^
Fear for safety because of crime	2.6 (2.5–2.8)	1.9 (1.5–2.3)^b^	2.4 (2.2–2.7)^b^	2.7 (2.4–3.0)^b^	3.3 (3.0–3.5)^b^
Not sure of physical activity benefits	2.6 (2.5–2.7)	2.0 (1.6–2.4)	2.4 (2.2–2.7)	2.6 (2.4–2.9)	3.1 (2.9–3.3)^b^
Community is not supportive of physical activity	2.6 (2.4–2.7)	2.0 (1.6–2.4)	2.4 (2.2–2.7)	2.6 (2.3–2.8)	3.2 (3.0–3.4)^b^
Fear for safety because of wild animals	2.5 (2.4–2.6)	2.0 (1.6–2.5)	2.3 (2.1–2.6)	2.5 (2.2–2.7)	3.1 (2.9–3.3)^b^
Hard to find time because of adult caregiving	2.5 (2.4–2.6)	2.0 (1.6–2.4)	2.3 (2.1–2.6)	2.5 (2.2–2.7)	3.1 (2.9–3.4)^b^

a Values are mean (95% CI). Rated on a scale of 1 (not at all a barrier) to 5 (very much a barrier).

b Significant difference (*P* < .05) detected by regression model.

### Regional and location differences

We saw no differences for any measured variables when comparing men who reported living in a small town versus those who reported living outside of a town. However, when comparing variables of interest by rural geographic regions (ie, Northeast, South, Midwest, West) in a linear regression model, we observed a significant difference in reported barriers to physical activity by region (Table 2). Although the order of barriers was mostly consistent by region, rural men in the West rated greater barriers to physical activity than men in other regions. Fear for safety because of COVID-19 was rated as the strongest barrier among men in the West.

## Discussion

### Interest in a physical activity intervention

Despite under-representation of US rural men in physical activity interventions, our nationwide sample reported being interested in receiving them. Previous interventions in rural communities have not been tailored to rural men (10,11). Although these men are interested in a physical activity program, the lack of success in previous interventions suggests a need for tailored interventions.

Rural men reported gaining fitness, increasing energy, and improving mental health as their top reasons for joining a physical activity program. In contrast, previous research showed that rural women ranked improved health, losing weight, and increased energy as their top reasons (15). Interventions among rural men that focused on weight loss have been successful in other studies (17), but how marketing may affect recruitment, including sampling bias, is unclear. Our results suggest promotional and recruitment materials that emphasize outcomes of fitness and increased energy instead of weight loss may appeal to more men. Future studies are needed on the effects of different marketing approaches on recruitment and retention. Intervention planners should consider their study population and the effect of marketing on that population’s perceptions of an intervention.

### Preferred intervention techniques

Men in our study reported a strong preference for remote delivery interventions, and mobile telephone applications were the highest-rated delivery method. This contrasts with findings among rural women who rated in-person meetings as their preferred delivery method (15). This difference may be due to men’s having a negative perception of certain exercise routines (18), resulting in a desire for privacy. Alternatively, this apparent preference may be a result of sampling differences. Cadmus-Bertram (15) collected data from mail surveys, whereas we used an online system. As a result, our sample may have included more technologically inclined men. In addition, the increased use of remote technology resulting from the COVID-19 pandemic could partially explain the differences. Our study was conducted during the pandemic and the Cadmus-Bertram study was conducted before the pandemic. In-person sessions have been used successfully in urban populations (19), but access to such sessions may be difficult for rural men given distances (20). Smartphones have been found to be an acceptable and feasible delivery method for other health interventions among rural men (17), adding further support for their use.

Our findings suggest that a physical activity intervention tailored to rural men should allow participation from home, provide specific instructions on how to exercise as opposed to the benefits of exercise, and teach how to incorporate exercise into a daily routine. Similarly, previous studies have reported that men desire straightforward information and prefer more purposeful physical activity such as active commuting, as opposed to planned exercise (19,20).

We found that the top barriers to physical activity among rural men were low motivation, cold weather, and tiredness. The lack of facilities was not listed as a top barrier is notable. Previous studies reported a lack of facilities and long commutes as major barriers among rural populations (21,22). Rural men may believe in-home resources are sufficient and may not need traditional exercise facilities as long as they have access to remotely delivered programs, but this possibility requires additional research. 

### Future directions

Although our findings suggest that rural men in the US are interested in participating in physical activity interventions and that their preference for remote delivery methods removes the barrier of facility availability, future studies are needed to develop and test physical activity interventions tailored to their needs and preferences. Given the heterogeneity of the rural male population, more work may be needed to apply these findings to specific groups (eg, men in various geographic regions). We observed differences in the strength of physical activity barriers when comparing rural men by geographic region. Interventions directed at rural men in Western regions may need to be designed differently to address that population’s strongest barriers, such as fear of exposure to COVID-19.

### Strengths and limitations

Our study has several strengths. It is the first study, to our knowledge, of what types of physical activity intervention rural US men want. The use of MTurk to recruit a nationwide sample of participants is novel and directly supported our goal of understanding rural men across all geographic regions.

Our study had several limitations. Although MTurk has been shown to generate data representative of the general population (23), our sample of MTurk workers could be more technologically savvy and more educated than the general population of rural men. The reported broadband access and use among our sample was similar to data collected among rural men by the Pew Research Center (24). However, our sample was more educated than the national average of rural men: 79.3% of our sample had a college degree compared with the national average of approximately 20% (25). Also, to ensure data quality, we followed recommended best practices (26) such as including only participants who passed the attention check and changing the survey settings to prevent automated responses and too-quick data entry. Sampling bias could also have been a limitation. Our study was advertised as a “survey on physical activity.” MTurk workers interested in physical activity may have been more likely to volunteer to take the survey. Because of the large heterogeneity of rural men, such as various cultures and environmental factors, our results may not be generalizable to all groups, particularly rural men with less education and men from minority groups. More research is needed to confirm the preferences we observed by studying specific subgroups of rural men, particularly those who were not well represented in our sample (ie, men in racial and ethnic minority groups and men with less education). Future studies could use more traditional methods to confirm our findings in specific populations, such as focus groups, interviews, and mail, telephone, or email surveys.

### Conclusion

Our findings suggest rural men are interested in physical activity programs despite their low representation in published studies of physical activity interventions. Rural men reported a preference for remotely delivered programs that could be carried out at home and as part of daily routines and that included straightforward demonstrations of physical activity. Further research is needed to determine whether physical activity interventions that incorporate our findings are effective for promoting physical activity behavior change among rural men.

## Appendix

**Appendix:** Supplemental Table. Interest Level in a Physical Activity Program Reported by a Nationwide Sample (N = 447) of Rural US Men, by Demographic Characteristics^a^

Demographic characteristic	All (N = 447)	Not at all interested (n = 23)	Not very interested (n = 49)	Somewhat interested (n = 118)	Interested (n = 150)	Very interested (n = 107)
**Hispanic**						
No	84.6	95.7	87.8	85.6	80.7	85.0
Yes	14.5	4.3	10.2	12.7	18.7	15.0
**Race **						
Black or African American	6.7	4.3	6.1	4.2	8.7	7.5
White	85.5	91.3	89.8	86.4	82.0	86.0
**Education**						
High school graduate	7.6	30.4	12.2	7.6	3.3	6.5
Some college	12.1	30.4	18.4	16.9	8.7	4.7
Trade, technical, or vocational training	2.9	4.3	8.2	4.2	1.3	0.9
College graduate	40.0	26.1	36.7	40.7	42.7	40.2
Some postgraduate work	8.9	4.3	4.1	10.2	10.0	9.3
Postgraduate degree	27.5	4.3	18.4	19.5	34.0	36.4
**Annual income, $**						
<24,999	12.1	17.4	12.2	8.5	12.0	15.0
25,000–49,999	22.6	47.8	36.7	22.0	18.0	17.8
50,000–74,999	22.4	26.1	26.5	25.4	22.7	15.9
75,000–99,999	25.3	4.3	12.2	28.8	28.7	27.1
100,000–149,999	14.6	4.3	8.2	14.4	15.3	18.7
≥150,000	2.9	0	4.1	0.8	3.3	4.7
**No. of children in household**						
0	31.8	69.6	49.0	41.5	20.7	20.6
1	33.8	13.0	18.4	36.4	36.7	38.3
2	29.3	13.0	16.3	21.2	38.7	34.6
≥3	4.7	4.3	16.3	0.8	3.3	5.6
**Marital status**						
Married	72.5	39.1	67.3	66.1	81.3	76.6
Never married	22.8	56.5	28.6	31.4	14.0	15.9
Divorced	2.2	4.3	4.1	1.7	2.7	0.9
**Self-reported health status**						
Excellent	40.3	17.4	8.2	33.9	44.7	49.5
Very good	34.3	39.1	42.9	29.7	30.7	29.9
Good	24.7	26.1	32.7	25.4	22.7	15.9
Poor	0.7	17.4	14.3	9.3	1.3	1.9
**Employment status**						
Fulltime	79.2	52.2	55.1	80.5	85.3	81.3
Looking for work	7.2	13.0	6.1	1.7	4.0	5.6
Student	3.4	0	2.0	1.7	2.0	0
Retired	3.4	17.4	14.3	2.5	0	0.9
**Occupational activity**						
Mostly sitting or standing	62.2	47.8	53.1	66.1	64.7	61.7
Mostly walking	19.9	4.3	26.5	21.2	20.0	18.7
Mostly heavy labor or physically demanding work	14.5	30.4	10.2	10.2	13.3	19.6

^a^ Values are percentage. Percentages may not add to 100 because of rounding.
